# Prehypertension is associated with increased carotid atherosclerotic plaque in the community population of Southern China

**DOI:** 10.1186/1471-2261-13-20

**Published:** 2013-03-19

**Authors:** Hua Hong, Hongxuan Wang, Huanquan Liao

**Affiliations:** 1 Department of Neurology, The First Affiliated Hospital, Sun Yat-sen University, No. 58, Zhongshan Road 2, Guangzhou 510080, P. R. China; 2 Department of Neurology, Sun Yat-sen Memorial Hospital, Sun Yat-sen University, No. 107, Yanjiang Road West, Guangzhou, 510120, P. R. China

**Keywords:** Carotid atherosclerosis, cIMT, Plaque, Hypertension, Prehypertension, Blood pressure

## Abstract

**Background:**

The proceeding of blood pressure (BP) from normal level to the hypertension has been found to be associated with increased cardiovascular events and multiple vascular risk factors. However, whether the process is associated with increased carotid atherosclerotic plaque per se or not is still unclear.

**Methods:**

Nine hundred and forty-two participants aged from 46 to 75 were enrolled from community population in Southern China. Their metabolic risk factors, carotid intima-media thickness (cIMT) and atherosclerotic plaque formation were analyzed and stratified by different blood pressure levels according to JNC-7 or ESH/ESC-2007 classification.

**Results:**

From low BP level to higher BP level, multiple metabolic risk factors increased linearly. Prehypertension in JNC-7 classification (or normal BP and high normal BP in ESH/ESC-2007 classification) was correlated with thicker cIMT and more plaque formation than normotension (or optimal BP) (*p <* 0.001). After adjusting multiple metabolic factors, the differences were still significant (*p <* 0.05). Furthermore, prehypertensive participants had a trend to be thicker carotid IMT (OR and its 95% CI: 1.65, 0.97-2.82, *p =* 0.067) and significantly higher carotid plaque occurrence (OR and its 95% CI: 2.36, 1.43-3.88, *p =* 0.001) than normotensive ones. However, there was no significant difference of cIMT and plaque formation between normal BP and high normal BP (*p >* 0.05). Plaque formation in prehypertension was as significant as that in hypertension.

**Conclusion:**

Prehypertension is associated with significantly increased carotid atherosclerotic plaque and is a primary stratify risk factor for carotid atherosclerosis which could cause ischemic stroke in middle-aged and elderly population in Southern China.

## Backgrounds

Hypertension is an important risk factor of atherosclerosis [1-3] and stroke [4,5]. There are more evidences showing that carotid atherosclerosis increases risk of ischemic stroke [6,7]. It is possible that carotid atherosclerosis could be one of the causal links between hypertension and ischemic stroke. For the past decades, several prospective studies indicates that a systolic/diastolic blood pressure above 115/75 mmHg is associated with increased risk of stroke [8], suggesting that the blood pressure status proceeding from normal to hypertension is a risk factor of stroke. The potential mechanism of this association is still unclear. Several studies have reported the blood pressure level from 120/80 mmHg to 139/89 mmHg is correlated with multiple vascular risk factors [9-11]. We assumed that the blood pressure status proceeding from normal to hypertension could cause carotid atherosclerosis. However, this hypothesis has not been proved, especially in Chinese population. Whether the normal blood pressure (BP) is associated with the same severity of carotid atherosclerosis as hypertension in Chinese population or not needs to be investigated. Furthermore, it is interesting to define the blood pressure level, which can stratify risk of carotid atherosclerosis in Chinese population.

In this study, we investigated the association of carotid atherosclerotic plaque formation and blood pressure level from normal (120/80 mmHg) to hypertension (140/90 mmHg) in middle aged and elderly community population in Southern China.

## Methods

### Study cohort

Study participants were enrolled from community populations in Guangzhou, China. Participants were enrolled by the Department of Neurology, the First Affiliated Hospital of Sun Yat-sen University from July 2008 to December 2008. Those who are aged from 46 to 75 with Chinese ethnic were eligible for the study. Those who had cardiovascular diseases more than six months ago and totally recovered without any persistent symptoms, sequelae or disabilities were not excluded in the study. However, those who had malignant tumors, acute or sub-acute symptomatic cardiovascular diseases (the period between the recovery from the diseases and the study recruitment was less than six months), and other critical illnesses were excluded. People who refused to complete necessary questionnaires were also excluded. The study protocol was approved by the Ethics Committee of the First Affiliated Hospital of Sun Yat-sen University, and all participants agreed on the written informed consent of the study.

### Medical risk factors and cigarette/alcohol intake

Previous histories of hypertension, diabetes, heart disease or stroke were defined if the subjects had been diagnosed with the disease in hospital before or had used medications regularly. Cigarette smoking was defined if the subjects had smoked more than one cigarette per day for more than half a year. Alcohol intake was defined if the subjects had alcohol consumption more than once a week for more than half a year.

### Physical and biological risk factors

Waist and hip circumference, weight and height were measured and waist-hip ratio (WHR) was calculated. Body mass index (BMI) was calculated as weight (kg) divided by the square of height (m^2^). Blood pressure was measured at least twice with a mercury sphygmomanometer after the participants had rested for no less than ten minutes in seat, and all measurements of the blood pressure were performed at the same time period in the morning by one well-trained investigator. If the two readings were different from each other for over 5 mmHg, a third measurement was performed after the participants had more rest in seat. The blood pressure of each participant was calculated as the average of the readings.

Blood samples for fasting blood glucose (FBG), total cholesterol (TC), triglyceride (TG), low-density lipoprotein cholesterol (LDL-C) and high-density lipoprotein cholesterol (HDL-C) were collected in the morning and then analyzed by the enzyme method in auto-analyzing machine. The coefficients of variance (CVs) of repeated measurements were 1.0% to 1.6%.

According to the definition of World Health Organization (WHO) in 1999 [12], central obesity, also called abdominal obesity, was defined as waist-to-hip ratio (WHR) > 0.90 for Chinese males or WHR > 0.85 for Chinese females. Participants with different levels of blood pressure were divided into several groups based on two main classifications, JNC-7 and ESH/ESC system. According to the JNC-7 in 2003 [13], hypertension (HT) is defined if systolic blood pressure (SBP) is ≥ 140 mmHg, diastolic blood pressure (DBP) is ≥ 90 mmHg, or if the patients have been diagnosed or had taken antihypertensive drugs ever before. Prehypertension (Pre-HT) is defined if the patients have not been diagnosed, or have not taken antihypertensive drugs and have a SBP between 120 to 139 mmHg and/or DBP between 80 to 89 mmHg. Normotension (NT) is defined if one has not been diagnosed, has not taken antihypertensive drugs before and has a SBP < 120 mmHg and DBP < 80 mmHg. According to the ESH/ESC in 2007 [14], hypertension and optimal blood pressure is defined the same as the hypertension and normotension in JNC-7, respectively. The normal BP is further divided into two groups; high normal BP, which has a SBP between 130 to 139 mmHg and/or DBP between 85 to 89 mmHg, and normal BP, which had a SBP between 120 to 129 mmHg and/or DBP between 80 to 84 mmHg.

### Ultrasound measurements of carotid IMT and plaques

**Figure 1:**
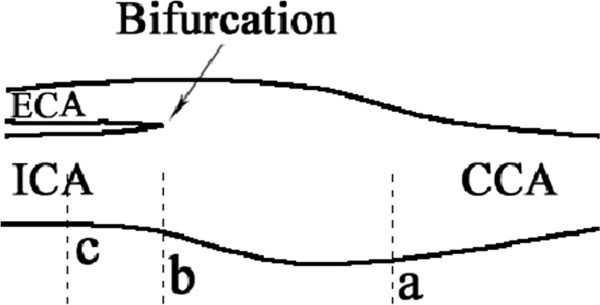
**The measurement of carotid IMT. **The point of carotid bifurcation is defined as the point (**b**). And the points of CCA and ICA are measured at point (**a**) and (**c**). Mean cIMT is calculated as the average cIMT of points **a** + **b** + **c**.

Carotid ultrasound measurements were performed with two B-mode ultrasound systems; APLIO XU equipped with a 7.5 MHz linear array transducer (Toshiba, USA) and HDI 5000 with a 5 ~ 12 MHz linear array transducer (Philips, USA). Subjects were examined in supine position with their necks extended. Measurements were taken in the diastolic phase in a proper direction for best visualization of the arteries. Intima-media thickness (IMT) was measured as the distance between the two parallel echogenic lines on the far wall of artery in longitudinal plane image frozen in the screen by electronic calipers [6]. IMT in three defined locations were measured bilaterally: common carotid (20 mm proximal to the bifurcation), carotid bifurcation and internal carotid (10 mm distal to bifurcation) (Figure 1). Mean IMT was calculated as the average of the six readings of bilateral carotid arteries. Plaque was defined as localized thickening of IMT ≥ 1.3 mm which did not uniformly involve the whole wall of carotid artery [1,15]. Quality controls were made by repeated scans on several randomly selected participants who were examined twice by two sonographers. The coefficient of variance (CV) of the mean IMT was 10.3%. The inter-observer difference was 0.08 ± 0.08 and the correlation of two readings was 0.661 (*p <* 0.001). The agreement of plaque occurrence was 88.1% and the kappa value was 0.738 (*p <* 0.001).

### Statistical methods

Continuous variables were described as unadjusted means ± standard deviations (SD) or means ± standard errors (SE) when the means were adjusted for age and/or sex. Dichotomized or categorized variables were described as numbers and unadjusted proportions. In the univariate analysis, unadjusted means were compared by student’s t-tests if the means were from two groups or by one-way ANOVA if the means were from more than two groups. In the multivariate analysis, adjusted means were compared by ANCOVA with different variables adjusted. Dichotomized or categorized variables were compared by Chi-square tests, and multivariate logistic regression was used for comparing different proportions when adjusting age, sex and other risk factors. Odds ratios (OR) were calculated by logistic model. All statistic analysis was calculated with SPSS 13.0 software system. A two-sided p-value of less than 0.05 was considered as statistically significant.

## Results

### Analysis of study cohort

Among 1025 residents who participated in our study, 942 residents completed all examinations and were included in the analysis. The 942 participants aged 57.2 ± 5.9 years old on average, which come from several provinces of Southern China, and they had lived and worked in Guangzhou for 45.1 ± 13.9 years on average. Of all the study population, 932 (98.9%) were the *Han* nationality. The proportion of previously diagnosed hypertension and diabetes mellitus in the study population was 28.0% and 9.6%, respectively, which were comparable with that in other randomized studies of Chinese population [16,17].

Among total 392 hypertensive participants, 234 (59.7%) had antihypertensive medicine daily, while the others had not taken antihypertensive drugs before because they were newly diagnosed (Table 1). Blood pressure of 309 residents fit in prehypertension group according to JNC-7 criteria. 241 residents have normotension blood pressure. None of residents in these two groups had taken antihypertensive medicine before. 66 (7.0%) participants had taken daily antidiabetic drugs and 42 (4.5%) participants had taken lipid-lowering medication (Table 1). Prehypertensive residents were further divided into 191 residents with normal BP and 118 residents with high normal BP according to ESH/ESC criteria (Table 2).

**Table 1:** Metabolic factors and carotid atherosclerosis in different blood pressure levels according to JNC-7

	**Normotension**	**Prehypertension**	**Hypertension**	***P *for trend**
N	241	309	392	----
Male/female	54/187	106/203 #	149/243 #	<0.001
Age, years	55.5 ± 5.0 §	56.4 ± 5.5 #§	58.9 ± 6.2 #	<0.001
WC, cm	77.1 ± 7.9 §	82.4 ± 8.5 #§	85.4 ± 9.1 #	<0.001
WHR	0.85 ± 0.06 §	0.88 ± 0.07 #§	0.90 ± 0.07 #	<0.001
BMI, kg/m^2^	21.8 ± 3.0 §	23.5 ± 3.1 #§	24.6 ± 3.3 #	<0.001
SBP, mmHg	107.4 ± 7.8 §	124.2 ± 6.4 #§	140.7 ± 15.2 #	<0.001
DBP, mmHg	69.2 ± 5.7 §	78.5 ± 5.4 #§	86.5 ± 10.3 #	<0.001
PP, mmHg	38.3 ± 7.1 §	45.7 ± 8.1 #§	54.2 ± 13.6 #	<0.001
FBG, mmol/L	5.0 ± 1.1 §	5.1 ± 0.9 §	5.4 ± 1.4 #	0.001
TC, mmol/L	5.19 ± 0.96	5.28 ± 0.97	5.25 ± 1.00	0.487
TG, mmol/L	1.41 ± 0.78 §	1.74 ± 1.10 #	1.98 ± 2.06 #	<0.001
LDL-C, mmol/L	3.53 ± 0.84	3.61 ± 0.88	3.65 ± 0.93	0.089
HDL-C, mmol/L	1.49 ± 0.34 §	1.39 ± 0.36 #§	1.31 ± 0.32 #	<0.001
DM, n (%)	13 (5.4) §	23 (7.4) §	54 (13.8) #	<0.001
CHD, n (%)	4 (1.7)	8 (2.6) §	38 (9.7) #	<0.001
IS, n (%)	0 (0.0)	4 (1.3)	14 (3.6) #	0.001
Smokers, n (%)	32 (13.3) §	59 (19.1)	82 (20.9) #	0.020
Alcohol, n (%)	13 (5.4)	25 (8.1)	32 (8.2)	0.230
WHO CO, n (%)	97 (40.2) §	179 (57.9) #§	275 (70.2) #	<0.001
Antihypertensive drugs, n (%)	0 (0.0)	0 (0.0) §	234 (59.7) #	<0.001
Antidiabetic drugs, n (%)	11 (4.6)	17 (5.5) §	38 (9.7) #	0.022
Lipid-lowering drugs, n (%)	5 (2.1%)	5 (1.6) §	32 (8.2) #	<0.001

*BP*, blood pressure; *WC*, waist circumference; *WHR*, waist-to-hip ratio; *BMI*, body mass index; *SBP*, systolic blood pressure; *DBP*, diastolic blood pressure; *PP*, pulse pressure; *FBG*, fasting blood glucose; *TC*, total cholesterol; *TG*, triglyceride; *LDL-C*, low-density lipoprotein cholesterol; *HDL-C*, high-density lipoprotein cholesterol; *DM*, diabetes mellitus; *CHD*, coronary heart diseases; *IS*, ischemic stroke; *WHO CO*, central obesity of WHO criteria; Values are unadjusted mean ± SD or number (%). P values are compared among the three/four groups by one-way ANOVA or *χ*^2 ^tests.

# versus Normotension, *p* < 0.05; § versus Hypertension, *p* < 0.05.

**Table 2:** Metabolic factors and carotid atherosclerosis in the population with normal BP and high normal BP according to ESH/ESC-2007

	**Normal BP**	**High normal BP**	***P *values**
N	191	118	----
Male/female	57/134	49/69	0.036
Age, years	56.1 ± 5.2	56.9 ± 5.8	0.042
WC, cm	81.5 ± 8.5	83.9 ± 8.5	0.938
WHR	0.88 ± 0.07	0.89 ± 0.06	0.575
BMI, kg/m^2^	23.2 ± 3.1	24.0 ± 2.9	0.568
SBP, mmHg	120.8 ± 4.2	129.7 ± 5.5	0.457
DBP, mmHg	77.2 ± 4.8	80.7 ± 5.6	0.386
PP, mmHg	43.7 ± 6.9	49.0 ± 8.8	0.121
FBG, mmol/L	5.1 ± 0.9	5.1 ± 0.9	0.597
TC, mmol/L	5.22 ± 0.97	5.38 ± 0.98	0.809
TG, mmol/L	1.70 ± 1.09	1.81 ± 1.12	0.930
LDL-C, mmol/L	3.60 ± 0.90	3.63 ± 0.84	0.793
HDL-C, mmol/L	1.40 ± 0.39	1.38 ± 0.31	0.362
DM, n (%)	12 (6.3)	11 (9.3)	0.323
CHD, n (%)	5 (2.6)	3 (2.5)	0.968
IS, n (%)	2 (1.0)	2 (1.7)	0.625
Smokers, n (%)	38 (19.9)	21 (17.8)	0.648
Alcohol, n (%)	16 (8.4)	9 (7.6)	0.814
WHO CO, n (%)	102 (53.2)	77 (65.3)	0.040
Antihypertensive drugs, n (%)	0 (0.0)	0 (0.0)	----
Antidiabetic drugs, n (%)	10 (5.2)	7 (5.9)	0.794
Lipid-lowing drugs, n (%)	4 (2.1%)	1 (0.8)	0.399
IMTmean, mm	0.76 ± 0.13	0.77 ± 0.14	0.596
Plaque (+), n (%)	43 (22.5)	34 (28.8)	0.213

*BP*, blood pressure; *WC*, waist circumference; *WHR*, waist-to-hip ratio; *BMI*, body mass index; *SBP*, systolic blood pressure; *DBP*, diastolic blood pressure; *PP*, pulse pressure; *FBG*, fasting blood glucose; *TC*, total cholesterol; *TG*, triglyceride; *LDL-C*, low-density lipoprotein cholesterol; *HDL-C*, high-density lipoprotein cholesterol; *DM*, diabetes mellitus; *CHD*, coronary heart diseases; *IS*, ischemic stroke; *WHO CO*, central obesity of WHO criteria; *IMTmean*, mean intima-media thickness of bilateral carotid arteries.

Values are unadjusted mean ± SD or number (percent). P values are compared between the two groups by student’s T-tests or *χ*^2^ tests.

### Metabolic factors and carotid atherosclerosis in different levels of blood pressure

Metabolic factors and carotid atherosclerosis in different levels of blood pressure according to JNC-7 and ESH/ESC classification were shown in Table 1 and Table 2, respectively. In both classifications, age, WC, WHR, BMI, SBP, DBP, PP, TG, FBG, the diabetes mellitus rates, coronary heart disease rates, ischemic stroke rates and cigarette smoker rates linearly increased from low BP subgroups to higher BP subgroups in both females and males (*p <* 0.05 for trend), while the levels of HDL-C had the reverse trend in both female and male (*p <* 0.05 for trend) (Table 1). The levels of TC and LDL-C and the alcohol intake rates did not have any significant changes in the BP subgroups in both female and male (*p >* 0.05) (Table 1).

**Figure 2:**
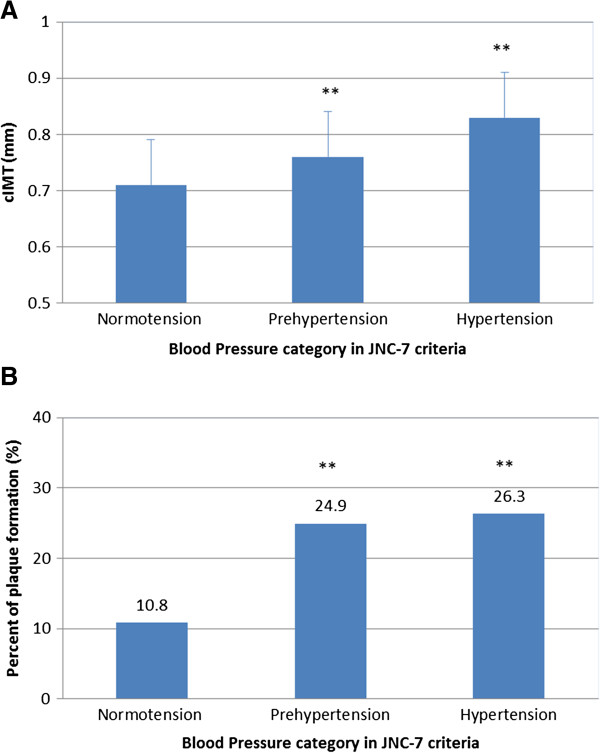
**Carotid IMT augment and the percentage of plaque formation in residents with normotension, prehypertesion, and hypertension in term of JNC-7 classification. **The blood pressures were divided into normotension, prehypertension, and hypertension groups. cIMT represents mean intima-media thickness of bilateral carotid arteries. ** versus normotension, *p* < 0.001 **A**. Mean carotid IMT (cIMT) in residents with normotension, prehypertesion, and hypertension in term of JNC-7 classification. **B**. Carotid plaque formation in residents with normotension, prehypertesion, and hypertension in term of JNC-7 classification.

**Figure 3:**
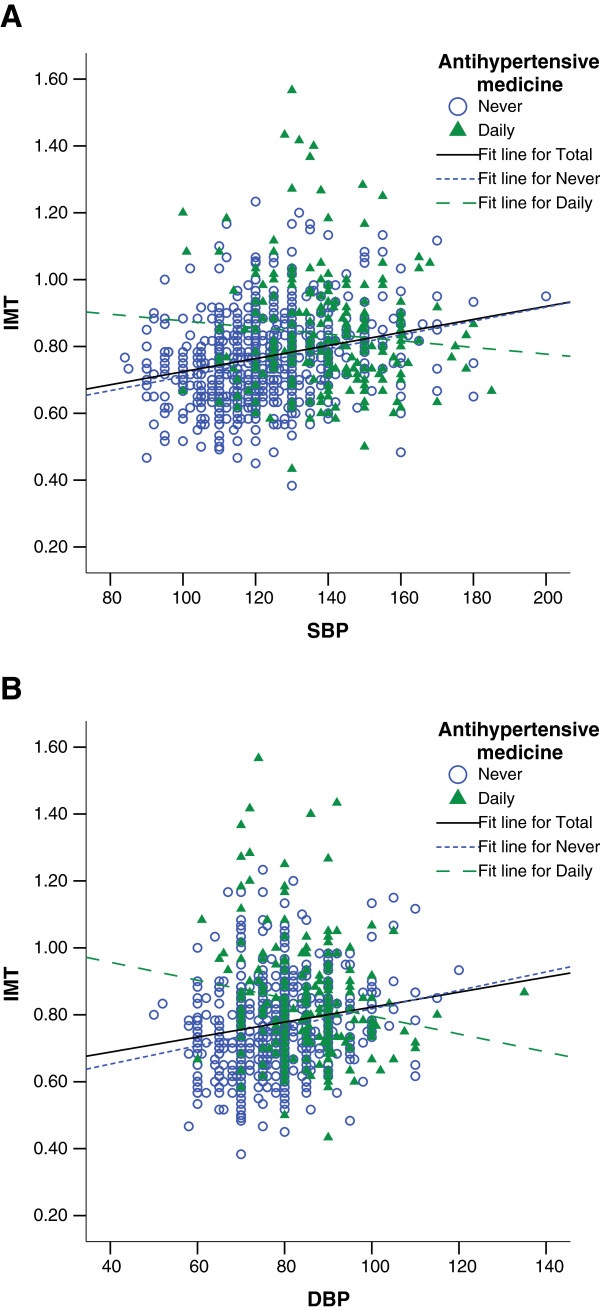
**Scatterplot of carotid IMT vs. systolic blood pressure (A) and diastolic blood pressure (B). **Antihypertensive drugs were never used or daily used in patient.

In the JNC-7 classification, prehypertensive and hypertensive residents accompanied with higher prevalence of central obesity than normotensive ones (57.9% and 70.2% vs. 40.2%, both *p <* 0.001) (Table 1). Pre-HT and HT had not only thicker mean cIMT in carotid arteries than normotensive ones (0.76 ± 0.13 mm and 0.83 ± 0.15 mm vs. 0.71 ± 0.11 mm, both *p <* 0.001) (Figure 2A), but also more occurrence of carotid plaque than normotensive ones (24.9% and 26.3% vs. 10.8%, *p <* 0.001) (Figure 2B), suggesting that both Pre-HT and HT are associated with cIMT augment and carotid plaque formation. In the participants who had never had antihypertensive medication, cIMT was positively associated with systolic and diastolic blood pressure (Figure 3A and 3B). Hypertensive residents had more prevalence of central obesity and had thicker mean cIMT than prehypertensive ones (*p =* 0.001 and *p <* 0.001) (Figure 2A). However, there was no difference in carotid plaque occurrence between hypertensive and prehypertensive residents (*p =* 0.683) (Figure 2B).

In the ESH/ESC classification (Table 2), residents with high normal BP had higher central obesity ratio than those with normal BP (*p =* 0.040). However, there was no difference in mean cIMT and plaque occurrence between normal BP and high normal BP (*p =* 0.681 and *p =* 0.213) (Table 2).

### Independent effect of prehypertension on carotid atherosclerosis

Given that there was no difference in carotid atherosclerosis between normal BP and high normal BP, we used prehypertension for further evaluation. As adjusting age, TG, HDL-C, FBG/diabetes, WC/central obesity and smoking status, prehypertensive participants had significantly thicker cIMT (*p <* 0.001) and more carotid plaque occurrence (*p <* 0.001) than normotensive participants (Table 3). Age, sex, hypertension, central obesity and smoking were independent determinants of thicker cIMT (Table 3). Hypertensive participants had significantly thicker cIMT than normotensive ones (OR and its 95% CI: 2.33, 1.40-3.87, *p =* 0.001), but prehypertensive participants had a trend to be with thicker cIMT than normotensive ones (OR and its 95% CI: 1.65, 0.97-2.82, *p =* 0.067). Age, prehypertension, central obesity and smoking were independent determinants of carotid plaque formation (Table 3). Prehypertensive participants had significantly more carotid plaque occurrence than normotensive ones (OR and its 95% CI: 2.36, 1.43-3.88, *p =* 0.001).

**Table 3:** Determinants of thicker carotid IMT and carotid plaque formation in the Logistic regression model

**Determinants**	**Thicker carotid IMT**	***P *value**	**Carotid plaque formation**	***P *value**
	**(OR and its 95% CI)**		**(OR and its 95% CI)**	
Age	1.11 (1.07, 1.14)	<0.001	1.10 (1.07, 1.13)	<0.001
Sex	1.86 (1.21, 2.86)	0.005	NS	NS
Central obesity	1.62 (1.11, 2.37)	0.013	1.55 (1.08, 2.22)	0.017
Smoking	1.99 (1.22, 3.24)	0.006	1.51 (1.02, 2.23)	0.040
Blood pressure levels				
Prehypertension	1.65 (0.97, 2.82)	0.067	2.36 (1.43, 3.88)	0.001
Hypertension	2.33 (1.40, 3.87)	0.001	1.88 (1.15, 3.07)	0.012

Thicker carotid IMT: upper quintile value of IMT.

*IMT*, intima-media thickness; *OR*, odds ratio; *CI*, confidence interval; *NS*, non-significant.

## Discussion

Consistent with previous studies in other populations [2,18-24], age and metabolic risk factors such as elevating blood pressure, body mass index, waist circumference, triglyceride, diabetes and heavy smoking in Chinese population positively correlated with carotid IMT and plaque (Table 3). However, which level of blood pressure would be the cut-off point to stratify significant status of atherosclerosis is not known. In this study, we found that the blood pressure level proceeding from normal to hypertension had been associated with significant carotid atherosclerotic plaque formation, independent of other vascular risk factors. The carotid atherosclerotic plaque formation in prehypertension was as severe as that in hypertension (Figure 2). Moreover, there is no difference of the carotid atherosclerotic plaque formation between normal BP and high normal BP in ESH/ESC-2007 criteria (Table 2), so prehypertension should be considered as the cut-off point to stratify the risk of carotid atherosclerosis which can cause ischemic stroke in middle-aged and elderly community population in Southern China. Prehypertensive residents never had antihypertensive medication before, thus the medication usage would not affect the association between carotid atherosclerotic plaque formation and blood pressure level proceeding from normal to hypertension.

Prehypertension and hypertension are associated with resemble risk of plaque occurrence after adjusting other metabolic risk factors in multivariate analysis (Table 3). However, the odds ratio of the association of prehypertension was slightly attenuated after multiple risk factors adjusted, indicating that some other risk factors such as obesity, triglyceride and smoking had synergistic effects on carotid plaque occurrence [1,23]. Consistent with previous hypothesis [1,25,26], IMT thickening and plaque formation in carotid arterie reflect different biological aspects of atherosclerosis. IMT thickening is mainly the result of hypertrophy of smooth muscles in the medial layer of carotid artery wall, while plaque formation is caused by pathological intimal thickening involving in the deposition of lipid. As a result, it has been hypothesized that IMT augment in the common carotid artery is strongly related to age and hypertension, while plaque formation or IMT in the carotid bulb or internal carotid artery is strongly related to hypercholesterolemia and smoking [1].

In recent years, several epidemiological studies have focused on the target-organ-damages in the blood pressure level which rises from 120/80 mmHg to 140/90 mmHg. Prehypertensive patients had been found to have higher CCA-IMT and larger left ventricular mass than their normotensive counterparts [27]. Here, we demonstrated similar findings in carotid IMT (Figure 2). We also found that prehypertensive residents had more occurrence of carotid plaque. Both normal BP and high normal BP had the same trends and there are no difference between these two subgroups. In previous report, Natali et al found that high normal BP, but not normal BP, was more accurate in identifying the linear trend of increasing metabolic risk factors, while prehypertension identified a dishomogeneous group of individuals [28]. The discordant results from Natali’s group and our group could be caused by different study populations. The Natali’s study was performed on a non-randomized sampled population who were free of hypertension, hyperlipidemia and diabetes mellitus, while our study was a cross-sectional analysis on a middle-aged and elderly Southern Chinese community population free of symptomatic cardiovascular diseases within 6 months. The underlying mechanism of the effect of prehypertension on the carotid atherosclerosis remains to be further determined.

Our data were collected from a community-based population who had lived and worked in Guangzhou for years. Of all the study population, the proportion of hypertension or diabetes mellitus in some age group of male and female was comparable with the randomized Chinese population in previous studies (Data not shown). It should be noted that our study population was not a randomized cohort and many community-based studies also encountered the same problem of non-randomized cohort. The study goal is to investigate the association among metabolic factors, carotid atherosclerosis and different blood pressure levels and would not be affected by non-randomized cohorts. In addition, the enough sample size and proper set of methodologies could make the analysis reliable (Table 1).

## Conclusions

In summary, prehypertension had been associated with significant carotid atherosclerotic plaque formation, independent of other vascular risk factors in middle-aged and elderly population in Southern China. Prehypertension could stratify the risk of carotid atherosclerosis and cardiovascular diseases, which cause ischemic stroke in Southern China. Our results suggest that prehypertension would be a warning status of higher risk of cardiovascular diseases. The population with prehypertension should be carefully monitored and life style modifications should be adapted to control BP and prevent the cardiovascular diseases and strokes.

## Competing interests

The authors declare that they have no competing interests.

## Authors’ contribution

HW and HL were responsible for data collection. HW was responsible for data analysis and preparation of the manuscript. HH gave advises and revised the manuscript. All authors contributed to data interpretation and critical revision of the manuscript. All authors read and approved the final manuscript.

## Authors’ information

First authors: Hua Hong and Hongxuan Wang.

## Pre-publication history

The pre-publication history for this paper can be accessed here:

http://www.biomedcentral.com/1471-2261/13/20/prepub
